# Bone Marrow Derived Extracellular Vesicles Activate Osteoclast Differentiation in Traumatic Brain Injury Induced Bone Loss

**DOI:** 10.3390/cells8010063

**Published:** 2019-01-17

**Authors:** Quante Singleton, Kumar Vaibhav, Molly Braun, Chandani Patel, Andrew Khayrullin, Bharati Mendhe, Byung R. Lee, Ravindra Kolhe, Helen Kaiser, Mohamed E. Awad, Tunde Fariyike, Ranya Elsayed, Mohammed Elsalanty, Carlos M. Isales, Yutao Liu, Mark W. Hamrick, Krishnan M. Dhandapani, Sadanand Fulzele

**Affiliations:** 1 Departments of Orthopedic Surgery, Augusta University, Augusta, GA 30912, USA; qsingleton@augusta.edu (Q.S.); chapatel@augusta.edu (C.P.); blee@augusta.edu (B.R.L.); cisales@augusta.edu (C.M.I.); 2 Department of Neurosurgery, Augusta University, Augusta, GA 30912, USA; kvaibhav@augusta.edu (K.V.); mobraun@augusta.edu (M.B.); bfariyike@augusta.edu (T.F.); 3 Cell Biology and Anatomy, Augusta University, Augusta, GA 30912, USA; akhayrullin@augusta.edu (A.K.); bmendhe@augusta.edu (B.M.); hkaiser@augusta.edu (H.K.); yutliu@augusta.edu (Y.L.); mhamrick@augusta.edu (M.W.H.); 4 Departments of Pathology, Augusta University, Augusta, GA 30912, USA; rkolhe@augusta.edu (R.K.); relsayed@augusta.edu (R.E.); melsalanty@augusta.edu (M.E.); 5 Department of Oral biology, Augusta University, Augusta, GA 30912, USA; moawad@augusta.edu; 6 Institute of Regenerative and Reparative Medicine, Georgia Regents University, Augusta, GA 30912, USA

**Keywords:** traumatic brain injury, bone loss, traumatic brain injury, extracellular vesicles, extracellular vesicles

## Abstract

Traumatic brain injury (TBI) is a major source of worldwide morbidity and mortality. Patients suffering from TBI exhibit a higher susceptibility to bone loss and an increased rate of bone fractures; however, the underlying mechanisms remain poorly defined. Herein, we observed significantly lower bone quality and elevated levels of inflammation in bone and bone marrow niche after controlled cortical impact-induced TBI in in vivo CD-1 mice. Further, we identified dysregulated NF-κB signaling, an established mediator of osteoclast differentiation and bone loss, within the bone marrow niche of TBI mice. Ex vivo studies revealed increased osteoclast differentiation in bone marrow-derived cells from TBI mice, as compared to sham injured mice. We also found bone marrow derived extracellular vesicles (EVs) from TBI mice enhanced the colony forming ability and osteoclast differentiation efficacy and activated NF-κB signaling genes in bone marrow-derived cells. Additionally, we showed that miRNA-1224 up-regulated in bone marrow-derived EVs cargo of TBI. Taken together, we provide evidence that TBI-induced inflammatory stress on bone and the bone marrow niche may activate NF-κB leading to accelerated bone loss. Targeted inhibition of these signaling pathways may reverse TBI-induced bone loss and reduce fracture rates.

## 1. Introduction

Traumatic brain injury (TBI) is a major cause of morbidity and mortality. TBI, which is defined as a blow or jolt to the head that produces permanent or temporary impairments in neurological function, affects individuals regardless of gender, ethnicity, age, and socio-economic status. Despite increased public awareness and improvements in safety measures, TBI contributes to nearly one-third of injury-related deaths [1,2,3], and millions of TBI survivors live with the long-term consequences of a prior TBI [4]. In contrast to other common neurological diseases, such as stroke and Alzheimer’s disease, TBI is more prevalent in younger populations, resulting in substantial loss of productive years and the need for lifelong assisted care. This burdens families and health care systems that provide cognitive, emotional, physical, and psychological support for TBI survivors. Altogether, TBI places an annual $76.5 billion burden on society [5,6].

In addition to the neurological consequences, TBI induces systemic immune changes that affect peripheral organs and worsen long-term quality of life [7,8,9,10,11,12]. Along these lines, TBI increases the risk of falls, fractures, osteopenia, and osteoporosis [13,14,15,16,17,18,19,20]. The increased risk for osteopenia and osteoporosis directly correlates with an elevated incidence of fractures and associated morbidity and mortality [21,22,23,24]. The mechanisms linking TBI with low bone mineral density and increased rates of fractures remain poorly defined and are likely multifactorial, with immobilization, epilepsy risk, anti-epileptic drugs, alcohol, and smoking as probable risk factors [13].

In this study, we investigated the effect of an isolated TBI on both macro and molecular bone changes. We hypothesized that inflammatory signaling in bone may represent a molecular link between TBI and increased bone resorption. In doing this, we isolated extracellular vesicles (EVs) from TBI bone marrow and showed their role in osteoclast differentiation. We also showed that bone marrow derived EVs following a TBI plays a vital role in bone marrow niche molecular signaling. Delineating the root cause of low bone mineral density and bone loss in patients suffering from TBI can further guide treatment and possibly reduce one of the major causes of morbidity and mortality in the patient population.

## 2. Materials and Methods

### 2.1. Controlled Cortical Impact

**Figure 1:**
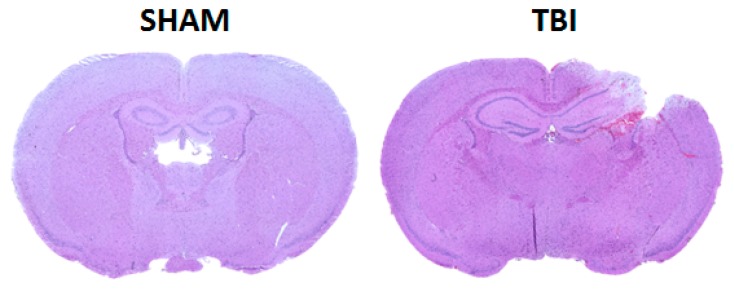
Representative cresyl violet-stained coronal brain sections from sham and TBI mice at 48 h.

The Institutional Animal Care and Use Committee (IACUC) at Augusta University approved all animal studies, in compliance with NIH guidelines (number: 2017-0838). Adult CD-1 male mice (*n* = 12–20) (Charles River, Wilmington, MA, USA) were subjected to a sham injury or moderate controlled cortical impact (CCI), as detailed by our laboratory [25]. Briefly, mice were anesthetized using 3% isoflurane, placed in a stereotaxic frame, and a craniotomy was made in the right parietal bone midway between bregma and lambda with the medial edge 1 mm lateral to the midline, leaving the dura intact. Mice were impacted at 3 m/s with a 100 ms dwell time and 3 mm depression using a 3 mm diameter convex tip (PinPoint PCI3000 Precision Cortical Impactor, Hatteras Instruments, Cary, NC, USA). Bone wax was used to seal the craniotomy, the incision was surgically stapled, and mice were placed in a clean warm cage until recovered. Sham-operated mice underwent the identical surgical procedures but were not impacted. The skin incision was closed and mice were allowed to recover in a clean, warm cage. Body temperature was maintained at 37 °C using a small animal temperature controller throughout all procedures (Kopf Instruments, Tujunga, CA, USA). Food and water were provided ad libitum. Histo-pathological analysis was performed on brain section after 48hrs using cresyl violet staining (Figure 1). Bones were collected for microCT analysis from sham-operated and TBI animals after 8 weeks.

### 2.2. Micro-Computed Tomography Analyses (µCT)

Micro-computed Tomography Analysis was performed (*n* = 12–20) as per our published method [26] post 8 weeks of sham-operated and TBI. For bone mineral density measurement and 3D morphometric analysis, 4% paraformaldehyde fixed femurs were scanned in a µCT system (Skyscan 1172; Skyscan, Aartlesaar, Belgium). Scanning was performed at an image pixel size of 14.59 μm. Reconstruction of the scanned images was done using a Skyscan Nrecon program. The reconstructed datasets were loaded into Skyscan CT-analyzer software for measurement of bone mineral density and 3D morphometric parameters. Distal femur was selected as region of interest; the bone mineral density was measured in the region of interest after calibration with hydroxyl apatite phantoms of known density.

### 2.3. Isolation of Bone Marrow Cells for Colony Forming and Osteoclast Differentiation Assay

The soft tissues were removed from the limbs with a sterile scalpel and the clean bones (*n* = 6) were transferred into a petri dish on ice. Both ends of the long bone (epiphysis) of the femur were cut to expose the bone marrow. The PBS was used to flush out the bone marrow and collected in a 15 mL tube. The bone marrow cell suspension was centrifuged at 300 g for 5 min, the supernatant was used for EVs isolation and the pellet was resuspended in culture medium. Bone marrow cells were cultured overnight in 100 mm tissue culture dishes in alpha-MEM media (5% heat inactivated FBS, 25 units/mL penicillin/streptomycin, and 400 mM L-Glutamine). After 24 h, non-adherent cells were collected, counted, and re-plated in 24-well plates at 2 × 10^3^ cells/cm^2^. Colony forming assay was performed by treating cells with alpha-MEM media (5% heat inactivated fetal bovine serum, 25 units/mL penicillin/streptomycin, and 400 mM L-Glutamine) containing 50 ng/mL M-CSF. For osteoclast differentiation cells were cultured in presence of 30 ng/mL macrophage colony-stimulating factor (M-CSF) and 50 ng/mL of RANKL for 4–6 days. The colony forming assay were stained with crystal violet and osteoclastogenesis cultures were stained for TRAP activity assay (Sigma; 387-A, Saint-Louis, MO, USA).

### 2.4. Tartrate-Resistant Acid Phosphatase Staining

Media was discarded from 24 cell culture plates and cells were washed twice with PBS and fixed as per manufactures protocol (tartrate of the Leukocyte Acid Phosphatase Assay kit, Sigma) for 30 min. After fixing, cells were washed twice with PBS, and then incubated with TRAP staining solution containing a mixture of Fast Garnet GBC, sodium nitrite, naphtol AS-BI phosphoric acid, acetate, and tartrate of the Leukocyte Acid Phosphatase Assay kit (Sigma) following the manufacturer’s instruction. TRAP-positive multinucleated cells were counted under a light microscope.

### 2.5. Isolation of RNA, Synthesis of cDNA, and Real-Time PCR

Total RNA was isolated from the tibia of mice (*n* = 6). For RNA isolation, the bone marrow cellular material was directly dissolved in Trizol whereas tibia bone particles were ground in liquid N2 with a pestle and mortar, and the powdered tissue was dissolved in Trizol. RNA was isolated using the Trizol method following the manufacturer’s instructions, and the quality of the RNA preparations was monitored by absorbance at 260 and 280 nm (Helios-Gamma, Thermo Spectronic, Rochester, NY, USA). The RNA was reverse-transcribed into complementary deoxyribonucleic acid (cDNA) using iScript reagents from Bio-Rad on a programmable thermal cycler (PCR-Sprint, Thermo Electron, Milford, MA, USA). 50 ng of cDNA was amplified in each real-time PCR using a Bio-Rad iCycler, ABgene reagents (Fisher scientific, Pittsburgh, PA, USA) using appropriate primers (Table 1). Glyceraldehyde-3-phosphate dehydrogenase (GAPDH) was used as the internal control for normalization.

### 2.6. Extracellular Vesicles Isolation from Bone Marrow

EVs were isolated using our published method [27,28,29]. Briefly, complete bone marrow (*n* = 6) content was dissolved into 500 uL PBS followed by centrifugation at 3000 RPM for 20 min to remove cell debris. The supernatant was collected and again centrifuged at 3000 RPM for 30 min to remove the remaining cell debris. Supernatant was collected and then Total Exosome Isolation Reagent (Life Technologies, Carlsbad, CA, USA) was used to isolate EVs as per manufacturer protocol. This protocol involved initial precipitation followed by centrifugation. After centrifugation, pellets were dissolved in 200 uL of phosphate-buffered saline (PBS) as EVs enriched fractions. The particle size and concentration of bone marrow derived EVs were measured using nanoparticle tracking analysis (NTA) with ZetaView (Particle Metrix, Meerbusch, Germany). Transmission electron microscopy and western blot was performed to validate our isolation approach. EM imaging of EVs preparations and western blot analysis was performed as described previously [27,28,29,30]. Isolated EVs were used for miRNA isolation and to perform functional studies. We have submitted all relevant data of our experiments to the EV-track knowledgebase (EV-TRACK ID: EV180076) [30].

### 2.7. Extracellular Vesicles Treatment

Bone marrow cells were cultured in 24-well plates and treated with sham and TBI bone marrow derived EVs (20 μg/mL) separately with 1% FBS (exosome free) media for 36 h. We pulled down BM derived-EVs from 8–10 sham and TBI separately to perform this experiment. IL-1, IL-6, TNFα, RELA, and Birc3 gene expressions were performed using real time PCR (Table 1)]. Colony forming assay and osteoclast differentiation assay was performed as mentioned above in the presence (20 μg/mL) or absence of EVs. The colony forming assay was stained with crystal violet and osteoclastogenesis cultures were stained for TRAP activity assay (Sigma; 387-A).

### 2.8. miRNA Isolation and Real Time PCR on Extracellular Vesicles 

MiRNA isolation and real time PCR was performed as per our published method [27,30]. In brief, miRNAs were isolated from EVs using miRNeasy Kit (Qiagen, Valencia, CA, USA) according to manufacturer’s protocol. The concentration of miRNA was determined using a NanoDrop spectrophotometer (Thermo Scientific, Wilmington, DE, USA). Real-time PCR was performed on miRNA-1224. We selected this miRNAs based on its role in NF-kb signaling [31] and osteoclast differentiation [32,33]. Two hundred nanograms of enriched miRNAs were converted into cDNA using miScript II RT Kit (from Qiagen). Fifty pictograms of cDNA were amplified in each qRT-PCR using SYBR Green I and miR specific primers (Qiagen). The real-time qRT-PCR was performed on a MyIQ machine (Bio-Rad, Hercules, CA, USA) with following cycling parameters: 95 °C for 10 min, then 40 cycles of 95 °C for 15 s, 60 °C for 30 s and 72 °C for 30 s. The average of RNU6 (RNA, U6 small nuclear 2) and SNORD (small nucleolar RNA, C/D box) was used as normalization reference genes for miRs. Relative expression of miRNA was evaluated by using the comparative CT method (ΔΔCt).

### 2.9. Statistics Analysis

GraphPad Prism 5 (La Jolla, CA, USA) was utilized to perform Unpaired Student’s *t*-test for microCT, real time PCR, and staining quantification. Differences between more than 2 groups were tested using one-way ANOVA. A *p* value of <0.05 was considered significant.

## 3. Results

### 3.1. Micro-Computed Tomography Analysis of Femur Bone

**Figure 2:**
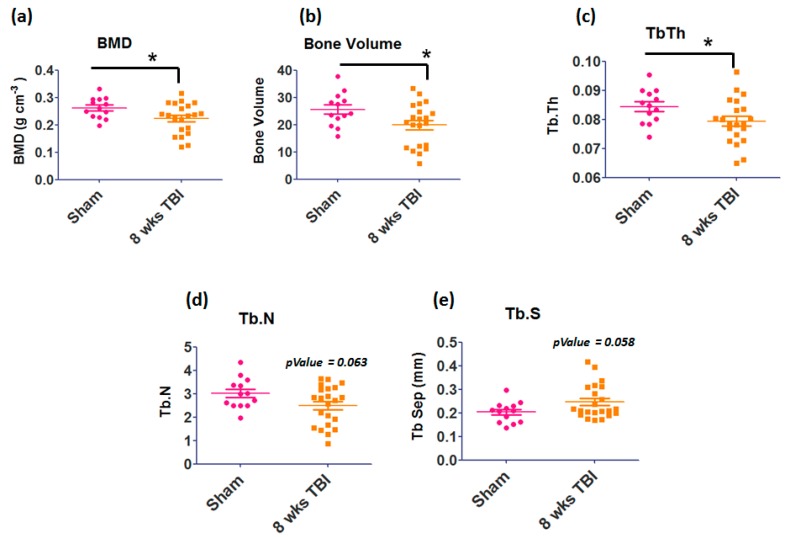
Effects of TBI on bone structural quality of femur measured by micro-computed tomography (μCT). (**a**) BMD, (**b**) bone volume, (**c**) tubercular thickness, (**d**) tubercular number were significantly decreased, and (**e**) tubercular separation was increased in the femurs of 8-week TBI mice compared to Sham. Results are means ± SD (*n* = 12–20). * Significant *p*-value 0.05.

Micro-computed tomography (microCT) was used to measure bone mineral density (BMD), bone volume/total volume (BV/TV), trabecular thickness (TbTh), and trabecular separation (Tb.Sp) in femurs from sham or TBI injured mice. Our data showed significant decreases in bone mineral density (*p* = 0.0365), bone volume (*p* = 0.0340), trabecular thickness (*p* = 0.0521), and trabecular number (*p* = 0.0630) in TBI mice compared to the controls (Figure 2). Furthermore, we found a trend toward increased (*p* = 0.058) trabecular separation in TBI mice.

### 3.2. TBI Decreased Bone Formation Markers and Increased Cytokines Expression in Bone

**Figure 3:**
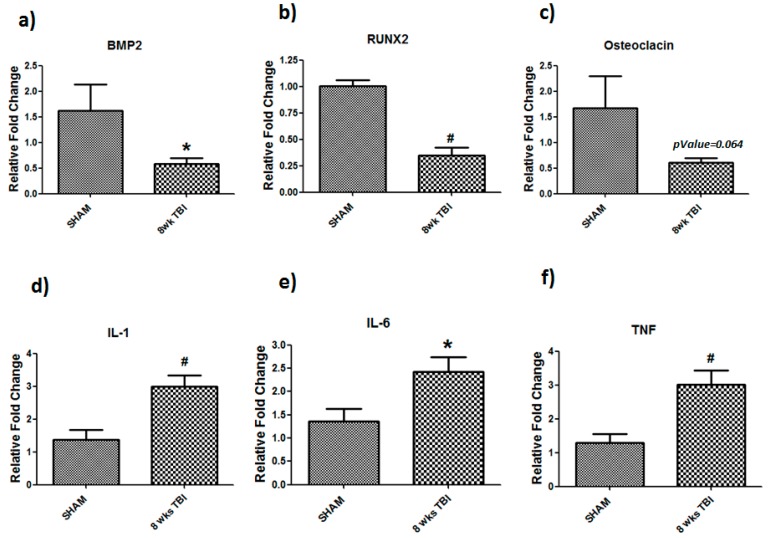
Dysregulation of bone markers and inflammatory genes in TBI bones. TBI animals showed decreased mRNA expression of (**a**) BMP2, (**b**) RUNX-2, and (**c**) osteocalcin and increased expression of (**d**) IL-1, (**e**) IL-6, and (**f**) TNF-α in 8-week TBI bones. After reverse transcription of total RNA, cDNA was amplified by quantitative real-time PCR. Data for each sample were normalized with GAPDH mRNA represented as the fold change in expression compared to sham mouse. Results are means ± SD (*n* = 6), Significant * *p* < 0.05, and # *p* < 0.01 determined by using student’s *t*-test.

Real-time PCR of bone-related markers and inflammatory genes was performed in bone chips derived from sham or TBI-injured mice at 8 weeks post-injury (Figure 3). All bone related genes showed down-regulation in TBI mice, as compared to sham injured mice. BMP2 (*p* = 0.05) and RUNX2 (*p* = 0.001) showed significant down-regulations in 8 weeks TBI bone whereas osteocalcin showed a trend of down-regulation (*p* = 0.064), as compared to sham (Figure 2c). In parallel to these changes, we observed chronic bone inflammation, as evidenced by increased expression of IL-1 (*p* = 0.001), IL-6 (*p* = 0.01), and TNF-α (*p* = 0.001) in 8 weeks TBI bones, as compared to sham (Figure 3d–f).

### 3.3. Elevated Chronic Inflammation and NF-κB Signaling Genes in Bone Marrow after TBI

**Figure 4:**
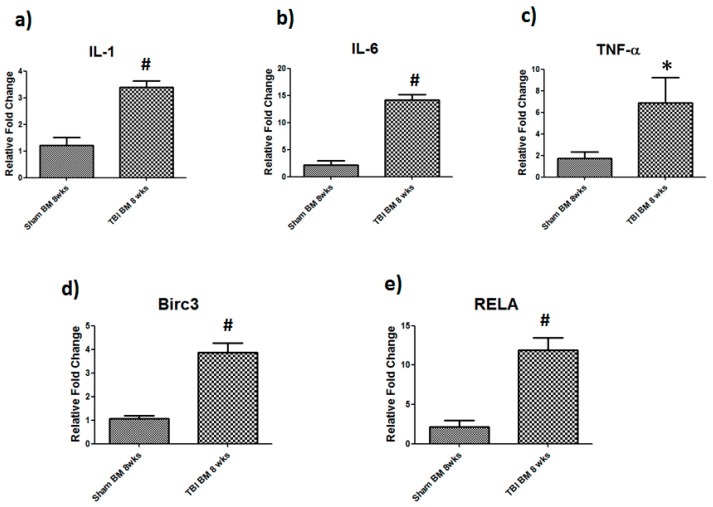
Elevated level of inflammatory and NF-kB signaling genes in TBI bone marrow. TBI mice showed increased mRNA expression of inflammatory genes (**a**) IL-1, (**b**) IL-6, (**c**) TNF-α and NF-kB signaling genes (**d**) Birc3 (**e**) RelA/p65 in 8-week TBI bone marrow. After reverse transcription of total RNA, cDNA was amplified by quantitative real-time PCR. Data for each sample were normalized with GAPDH mRNA represented as the fold change in expression compared to sham mouse. Results are means ± SD (*n* = 6), Significant * *p* < 0.05, and # *p* < 0.01 determined by using student’s *t*-test.

As bone marrow produces hematopoietic and mesenchymal stem cells, we next performed real-time PCR on selected inflammatory and NF-κB signaling genes in bone marrow. We found that both inflammatory and NF-κB signaling genes were dysregulated following TBI (Figure 4). Specifically, IL-1 was increased 3-fold (*p* = 0.001) whereas TNF-α was up-regulated six-fold (*p* = 0.04) after TBI, as compared to sham-operated mice. IL-6 showed the most profound up-regulation with a ten-fold increase, as compared to sham group. We also observed an increase NF-κB signaling genes (Birc3 and RelA/p65 genes). Birc3 gene increased four-fold (*p* = 0.001), whereas RelA/p65 exhibited a ten-fold increase after TBI (*p* = 0.001).

### 3.4. TBI Affects Colony Forming Unit (CFU) Efficiency and Osteoclast Differentiation of Bone Marrow Cells

**Figure 5:**
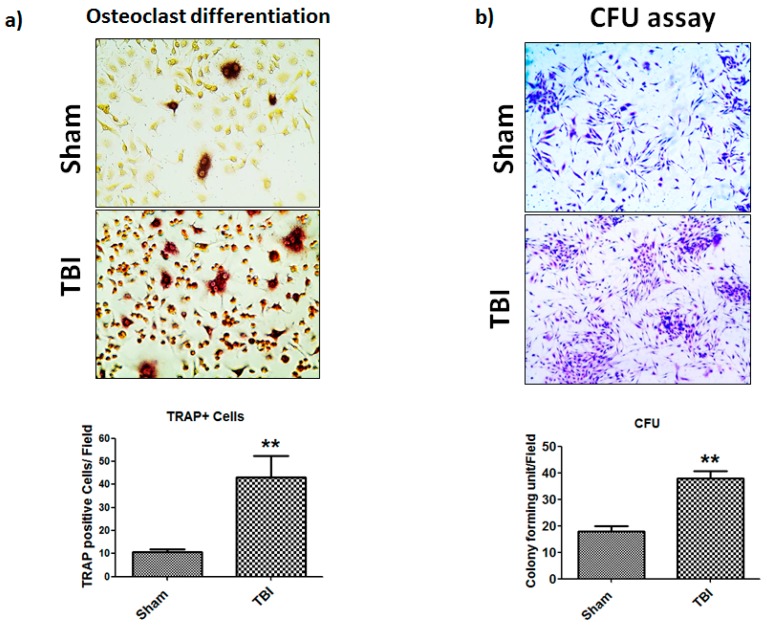
Effect of TBI on osteoclast differentiation and colony forming unit (CFU) on bone marrow cells. (**a**) Mouse primary bone marrow cells were cultured with RANKL (100 ng/mL) and M-CSF (30 ng/mL) for 4 days followed by TRAP staining. After TRAP staining, TRAP + multinuclear cells (TRAP + MNCs) with more than three nuclei were scored as osteoclasts. (**b**) Colony forming assay was performed, stained with crystal violet, and colonies were counted * *p* < 0.05 and ** *p* < 0.01 compared with vehicle-treated control.

Colony forming cells are one of the important pre-osteoclast cells which differentiate into osteoclasts. Thus, we next hypothesized that the elevated level of bone loss observed by microCT after TBI was due to increased colony forming activity and osteoclast differentiation efficiency by bone marrow cells after TBI. To test this hypothesis, isolated bone marrow cells from sham or TBI mice were cultured in the presence of macrophage colony-stimulating factor (M-CSF) for CFU and osteoclast media for osteoclast differentiation. TBI-derived bone marrow cells exhibited significantly higher (*p* = 0.01) CFU and cell proliferation efficiency (Figure 5b). The osteoclast differentiation assay also showed similar findings. We found that TBI derived bone marrow cells have significantly (*p* = 0.01) higher TRAP positive multinucleated cells compared to sham group (Figure 5a).

### 3.5. Extracellular Vesicle Isolation and Characterization

**Figure 6:**
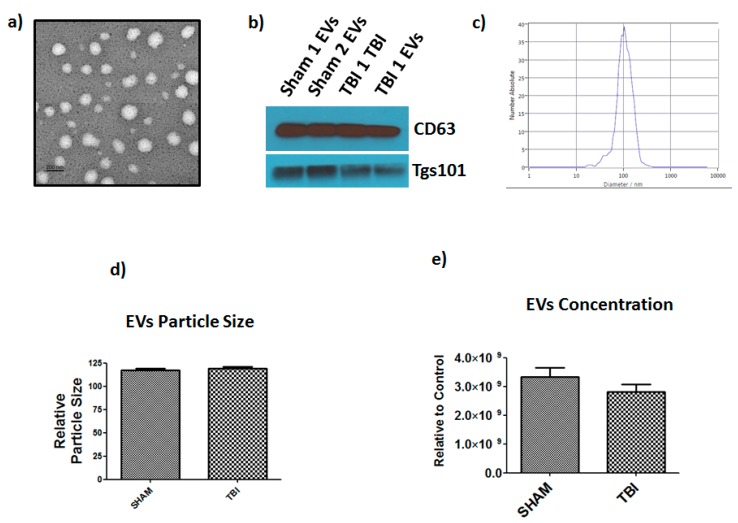
Characterization of TBI bone marrow derived EVs. (**a**) Transmission electron microscope images of EVs. (**b**) Western blot demonstrating the expression of CD63, and TSG101 in BM derived EVs. (**c**) Particle size distribution is consistent with size range of EVs (average size 100 nm), measured by ZetaView^®^ Particle Tracking Analyzer. No significant change in (**d**) particle size and (**e**) concentration in TBI and sham bone marrow derived EVs (*n* = 6).

We isolated EVs from sham and TBI mice bone marrow using precipitation and centrifugation method as per our published method [27,28,29]. Electron micrographs showed that the isolated EV particles are round shaped vesicles (Figure 6a) and western blot (Figure 6b) analysis showed band of exosome markers Tsg101, and CD63. Previously, we showed immuno-gold staining for CD-9, and CD-63 on EVs isolated from mouse bone marrow [29]. Nanoparticle tracking analysis showed that vesicles isolated from bone marrow are in the ~100 nm diameter size range, consistent with the known size of EVs [27,28,29]. We did not find any significant changes in size or concentration of bone marrow derived EVs of TBI (Figure 6d). 

### 3.6. EVs Derived from TBI Bone Marrow Enhance Osteoclast Differentiation of Bone Marrow Cells

**Figure 7:**
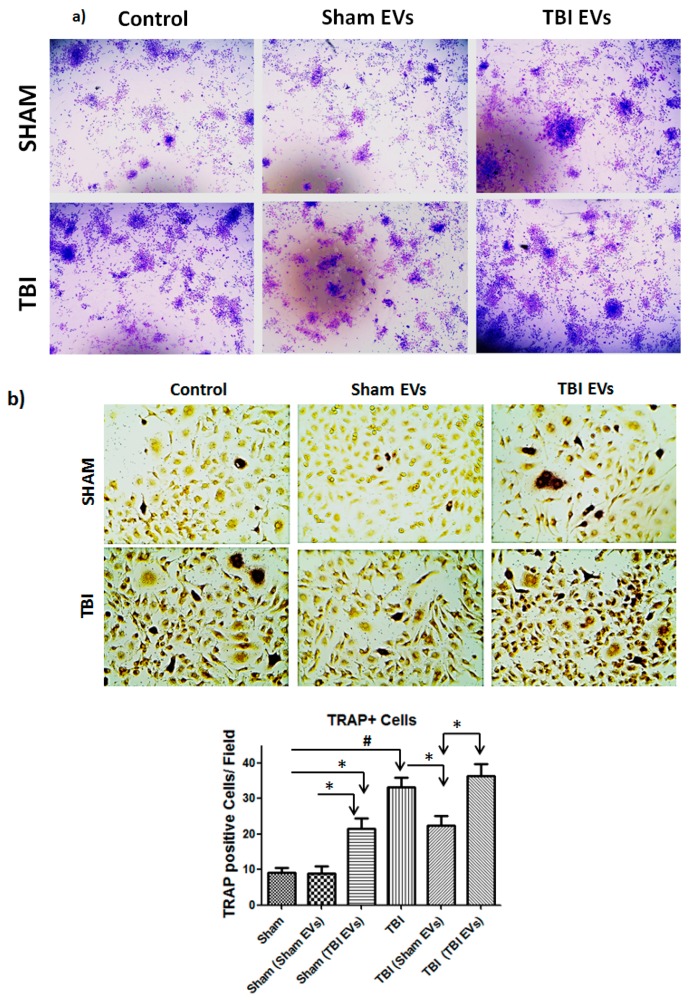
Effect of bone marrow derived EVs from TBI on colony forming unit (CFU) and osteoclast differentiation of bone marrow cells. (**a**) Colony forming assay were performed with M-CSF (30 ng/mL) in the presence or absence of sham/TBI derived EVs for 4 days, stained with crystal violet staining, and colonies were counted. (**b**) Mouse primary bone marrow cells were cultured with RANKL (100 ng/mL) and M-CSF (30 ng/mL) in the presence or absence of sham/TBI derived EVs for 6 days followed by TRAP staining. After TRAP staining, TRAP + multinuclear cells (TRAP + MNCs) with more than three nuclei were scored as osteoclasts (Significant * *p* < 0.01, # *p* < 0.001).

Our data suggests the increase in TRAP positive multinucleated cells formation in the TBI bone marrow cells is due to changes in bone marrow microenvironment. We hypothesized that increased osteoclast differentiation of TBI bone marrow cells are partially due to extracellular vesicles. To test this hypothesis, bone marrow cells from sham and TBI mice were cultured in the presence of EVs derived from sham or TBI bone marrow. We found that TBI derived EVs significantly affected colony forming units as well as osteoclast differentiation efficiency of normal bone marrow cells (Figure 7). Furthermore, EVs derived from sham bone marrow partially prevented colony forming units and osteoclast differentiation efficiency of TBI bone marrow cells (Figure 7b).

### 3.7. TBI-Derived EVs Isolated from Bone Marrow Regulate Inflammatory and NF-κB Signaling

**Figure 8:**
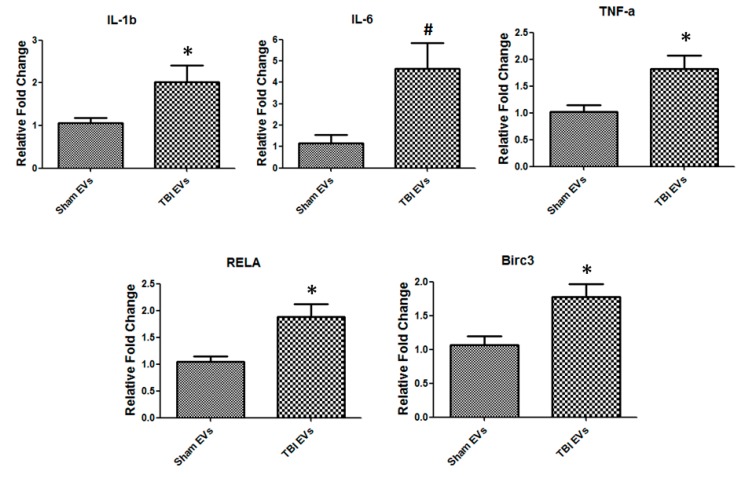
TBI derived bone marrow EVs regulate inflammatory and NF-kB signaling gene expression. Bone marrow cells treated with sham and TBI derived EVs at concentrations of 20 µg/mL for 36 h followed by RT-PCR, IL-1, IL-6II, TNF-a, and RelA/p65 and Birc3 (*n* = 6, * *p* < 0.05, # *p* < 0.01).

To gain further insight into the role of EVs in osteoclast differentiation, normal bone marrow cells were treated with EVs isolated from sham and TBI bone marrow. We found that EVs regulate inflammatory and NF-κB signaling of bone marrow cells. Our results showed a significant increase in IL-1 (*p* = 0.026) and TNF-α (*p* = 0.042) in bone marrow cells. IL-6 (*p* = 0.018) had the highest up-regulation compared to IL-1 and TNF-α. Furthermore, NF-κB signaling genes Birc3 (*p* = 0.0054) and RelA/p65 (*p* = 0.0028) were significantly up-regulated compared to control (Figure 8).

### 3.8. The miRNA-1224 Cargo Changed in TBI-Derived EVs

**Figure 9:**
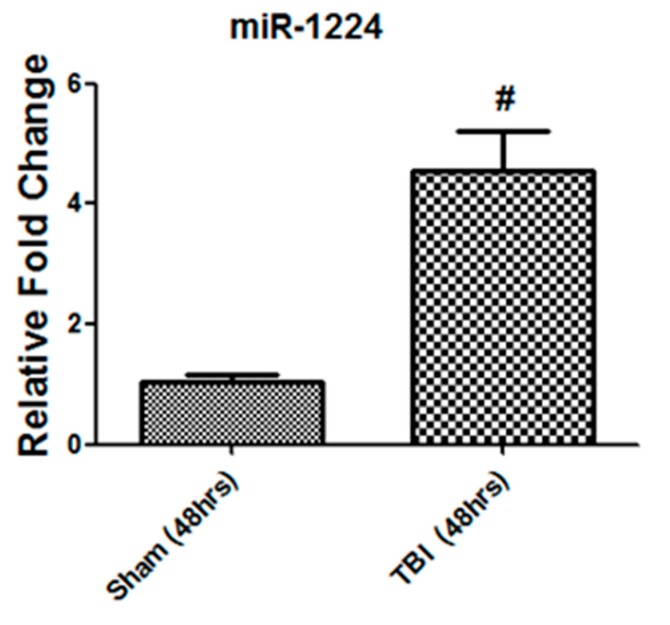
TBI alters the miRNA-1224 content in bone marrow-derived EVs. Real-time PCR showing change in miRNA-1224 expression in 48 h TBI bone marrow-derived EVs cargo (*n* = 8, # *p* < 0.001).

MiRNA-1224 is known for its role in NF-kb activation [31] and osteoclast differentiation of RAW264.7 cells [32,33]. Our functional assayed showed that TBI bone marrow-derived EVs activate NF-kb signaling and osteoclast differentiation of bone marrow cells (Figure 5 and Figure 7). We hypothesized that miRNA-1224 might be dysregulated in TBI bone marrow-derived EVs. To investigate this, we isolated miRNA and perform real time PCR on miRNA-1224. Real time data showed significant (*p*-value = 0.001) up-regulation (~4 fold) of miRNA-1224 in 48h TBI bone marrow-derived EVs compare to sham (Figure 9). 

## 4. Discussion

TBIs induce chronic, broad sequelae that reduce long-term quality of life. Clinical data suggest a strong correlation between TBI and dysfunctions in autonomic regulation, neuroendocrine function, and psychiatric stability [1,5,6,7,8]. Moreover, recent studies suggest that increased fracture rates are another long-term consequence of TBI [13,14,15,16,17,18,19,20]. The combination of an increased fall risk and low bone mineral density post-TBI has been associated with increased fracture rates [13,14,15,16,17,18,19,20]. In this study, we used an established pre-clinical model of focal TBI in mice. We found that a single, isolated head injury decreased bone mineral density and increased bone loss. Furthermore, our data also demonstrated decline in bone markers such as BMP2, RUNX2, and osteocalcin in bone and elevated levels of pro-inflammatory cytokines in bone marrow niche/environment after TBI. It is well established that traumatic injury to the brain produces inflammatory responses in the bloodstream and peripheral organs [34]. Ours is the first study to demonstrate elevated levels of pro-inflammatory cytokines in the bone marrow niche/environment. The systemic production of pro-inflammatory cytokines in the bone marrow, bloodstream, and peripheral organs may play a vital role in secondary complications of TBI. Recent TBI studies in mice have shown similar damaging inflammatory cascades outside the central nervous system such as in the bloodstream [35], liver [34], kidney [36], and other organs. Hayakata et al. (2004) reported elevated levels of pro-inflammatory cytokines in serum in the acute setting of a TBI within the first six hours, post injury [37].

In normal physiological conditions, there is a balance between the activity of bone resorbing cells (osteoclasts) and bone forming cells (osteoblasts); however, this homeostasis may be disrupted under pathological conditions, leading to bone loss. Consistent with our findings showing a reduction in bone mineral density and increased bone loss, our in vitro data suggest bone marrow hematopoietic cells derived from TBI mice enhanced osteoclast activity, as compared to bone marrow from sham-injured mice. Beyond the demonstration of elevated levels of osteoclast differentiation after TBI, bone marrow from TBI mice increased colony formation, suggesting TBI creates an ideal microenvironment for osteoclast differentiation. Although the precise mechanisms underlying these effects remain undefined, oxidative stress and inflammation contribute toward post-menopausal and age-dependent bone loss [38,39]. In particular, bone resorption and differentiation of osteoclast precursors to mature cells is regulated by the pro-inflammatory transcription factors, NF-κB, and RANKL [40,41]. Of note, we reported increased chronic inflammatory activation, involving the mobilization of bone marrow derived immune cells, within both blood and brain following a TBI [25,42,43,44,45]. Consistent with these findings, the key NFκB genes, RelA/p65, and Birc3, were dysregulated in the bone marrow niche after TBI. Moreover, Vaira et al. (2008) reported that RelA/p65 promotes osteoclast differentiation by blocking RANKL induced apoptosis whereas knockdown of RelA in the hematopoietic compartment blocked osteoclastogenic response to RANKL and protected against arthritis-induced osteolysis [40]. Thus, TBI may create a chronic, pro-inflammatory environment within the bone marrow that contributes to progressive bone loss.

Different cell types within the bone marrow cavity communicate via the release of extracellular vesicles (EVs), which are ~100 nm diameter packaged vesicles containing specific proteins, lipids, factors, and/or genetic material. Recent studies suggested that bone marrow- and blood-derived exosomes regulate osteoblastic and osteoclastic differentiation in various musculoskeletal disease models [46,47,48]. As we similarly demonstrated that human synovial fluid-derived EVs play vital role in the pathophysiology of osteoarthritis [27], we herein explored whether EVs contribute to osteoclast differentiation efficiency after TBI. To answer this important question, we cultured bone marrow hematopoietic cells derived from sham-injured mice in the presence of bone marrow derived EVs from TBI mice. Interestingly, bone marrow derived EVs from TBI mice increased both osteoclast differentiation and colony forming cells in sham-derived bone marrow cells. Furthermore, we demonstrated that sham bone marrow derived EVs partially prevented osteoclast differentiation efficiency of TBI bone marrow cells. Moreover, bone marrow derived EVs isolated from TBI mice elevated pro-inflammatory cytokines and dysregulated NF-κB signaling genes in bone marrow cells. Our findings in the context of TBI are in line with a report showing EVs derived from various body fluids, including amniotic fluid, liver cirrhosis ascites, and malignant ascites of ovarian cancer patients, activate inflammatory cytokines in monocytic cells via NF-κB signaling [49].

EVs miRNAs cargo plays important role in normal cellular and pathological conditions [27,29]. Our published studies demonstrated that bone marrow-derived EVs miRNA cargo change with age [29]. Previously it has been reported that miR-1224 regulate NF-kb activity in RAW264.7 cells [31] and play important role in osteoclast differentiation [32,33]. Based on these studies [31,32,33] and our findings that TBI derived EVs activate NF-kb and osteoclast differentiation (Figure 5 and Figure 7), we hypothesized that TBI bone marrow-derived EVs miRNA-1224 cargo might be affected. This is indeed the case; we found that miR-1224 elevated in TBI bone marrow-derived EVs. Niu et al. reported that miR-1224 mimic transfection to RAW264.7 cells increase the basal NF-kb activity and Kagiya et al. group reported elevated level of miR-1224 expression during osteoclast differentiation of RAW264.7 cells. We speculate that elevated level of miR-1224 in EVs might play important role in TBI dependent NF-kb activation and osteoclast differentiation in bone marrow. Further studies are needed to demonstrate direct relationship between EVs miR-1224 cargo and NF-kb activation/osteoclast differentiation.

Our well-established, pre-clinical TBI model produces a highly reproducible focal TBI; however, clinical TBI is a heterogeneous injury that may not be perfectly mimicked by any single rodent model. Thus, confirmation of our findings using other TBI models, such as lateral fluid percussion, and higher order species (e.g., porcine models) may be warranted prior to clinical translation. Our model used herein also produces a moderate-severe injury; thus, it would be interesting to determine whether similar effects are observed on bone density after a single and/or repetitive mild TBI. These later studies may have direct relevance to athletes in contact sports and military personnel that are at risk of TBI. A potential caveat is the use of young, otherwise healthy male mice. While necessary to limit the scope of this proof of concept study, our studies do not consider the potential influence of common comorbidities that may influence bone physiology, including age and sex. Furthermore, we only assessed EVs effects in in vitro cultures of bone marrow cell differentiation to osteoclasts. Further studies are needed to determine whether TBI derived exosomes affect the osteogenic differentiation ability of mesenchymal stromal cells. In addition, we did not elucidate in detail which cargo (protein, miRNA) of exosomes is directly responsible for the increased pro-inflammatory cytokine production and osteoclast differentiation. Future studies are needed to investigate the EVs cargo and their role in TBI-induced bone loss.

Taken together, our study raises the interesting possibility that TBI fosters a chronic pro-inflammatory state within the bone marrow niche, culminating in increased bone resorption. Future work by our group will elucidate the source of EVs in bone marrow to determine whether EVs are locally released or transported from the injury site. We also will identify the cargo of TBI-derived EVs to further advance therapeutic development and the clinical translation of targeted therapies to prevent bone loss after TBI.

## Figures and Tables

**Table 1:** Nucleotide sequences of mouse primers used for RT-PCR.

Gene	Primer	Reference/Accession Number
GAPDH	CAT GGC CTC CAA GGA GTA AGA GAG GGA GAT GCT CAG TGT TGG	M32599
BMP-2	TGT TTG GCC TGA AGC AGA GA TGA GTG CCT GCG GTA CAG AT	NM_007553.2
RUNX-2	GGA AAG GCA CTG ACT GAC CTA ACA AAT TCT AAG CTT GGG AGG A	NM_009820
Osteocalcin	ATT TAG GAC CTG TGC TGC CCT A GGA GCT GCT GTG ACA TCC ATA C	U11542.1
IL-6	TAG TCC TTC CTA CCC CAA TTT CC TTG GTC CTT AGC CAC TCC TTC	NM_031168.1|
IL-1	GCA CCT TAC ACC TAC CAG AGT AAA CTT CTG CCT GAC GAG CTT	NM_031168.1|
TNF	CCC TCA CAC TCA GAT CAT CTT CT GTC ACG ACG TGG GCT ACA G	NM_013693.2|
RELA	GGA GGA TGC CTC CTG CAA AC TGT AGT GGA AGC CCT GTC CT	AF199371
Birc3	ACG CAG CAA TCG TGC ATT TTG CCT ATA ACG AGG TCA CTG ACG G	AJ401388

## Abbreviations

TBI	Traumatic brain injury
EVs	Extracellular vesicles
M-CSF	Macrophage colony-stimulating factor
GAPDH	Glyceraldehyde-3-phosphate dehydrogenase
BMP2	Bone morphogenetic protein 2
RUNX2	Runt-related transcription factor 2
TRAP	Tartrate-resistant acid phosphatase
